# Complete Genome Sequence of Persistent Cystic Fibrosis Isolate *Pseudomonas aeruginosa* Strain RP73

**DOI:** 10.1128/genomeA.00568-13

**Published:** 2013-08-01

**Authors:** Julie Jeukens, Brian Boyle, Irene Bianconi, Irena Kukavica-Ibrulj, Burkhard Tümmler, Alessandra Bragonzi, Roger C. Levesque

**Affiliations:** Institut de Biologie Intégrative et des Systèmes (IBIS), Université Laval, Quebec, Canadaa; San Raffaele Scientific Institute, Division of Immunology, Transplantation and Infectious Diseases, Milan, Italyb; Clinical Research Group, Hanover Medical School, Hanover, Germanyc

## Abstract

*Pseudomonas aeruginosa* can establish lifelong chronic airway infections in cystic fibrosis (CF) patients. However, the genetic features associated with long-term persistence in the lung are not understood. We sequenced the genome of *P. aeruginosa* strain RP73, which was isolated after 16.9 years of chronic lung infection in a CF patient.

## GENOME ANNOUNCEMENT

The Gram-negative bacterium *Pseudomonas aeruginosa* is a clinically relevant opportunistic pathogen highly resistant to most classes of antibiotics (1). It is the leading cause of chronic long-term lung infections and death in cystic fibrosis (CF) individuals. Following an initial period of intermittent and acute infections, *P. aeruginosa* establishes a persistent lifestyle in CF airways (2). Long-term colonization of the host is maintained by patho-adaptive lineages, which are presumably clonal from the initially acquired strain (3). Adaptive and loss-of-function mutations are frequent in virulence genes, which are essential for the initiation of infection and transient-to-long-term persistence in CF airways (4, 5). We selected the multidrug resistant nonmucoid *P. aeruginosa* strain RP73 as a model to investigate the genomic basis for long-term persistence in CF lung infections. RP73 was isolated 16.9 years after the onset of infection in a CF patient from the Hannover cohort (6). RP73 can establish and maintain long-term lung infection in a murine model of chronic pneumonia (7–9) with histopathological lesions similar to those found in CF patients (10). Hence, this strain is particularly well suited for comparative genomics with other CF strains, the study of *P. aeruginosa* pathogenesis, and the evaluation of novel antimicrobial therapies.

Following isolation, strain RP73 was stored at –80°C in Tryptic soy broth (TSB) with 30% glycerol. Genomic DNA was isolated from an overnight culture using the DNeasy blood and tissue kit (Qiagen). Whole-genome shotgun DNA sequencing was performed using the Roche 454 pyrosequencing method on the Genome Sequencer FLX system with titanium chemistry at the Plateforme d’analyses génomiques of the Institut de Biologie Intégrative et des Systèmes (Université Laval). In total, 319,835,813 nucleotides were analyzed using the gsAssembler module of Newbler v2.5.3. A total of 68 contigs were produced; 40 were larger than 500 nucleotides. Genome finishing was performed using Consed v. 20 (11). Automated annotation was performed with xbase (12) and the NCBI Prokaryotic Genome Annotation Pipeline (PGAAP) using *P. aeruginosa* PAO1 as a reference genome.

The assembled RP73 genome consists of a single circular chromosome of 6,342,034 bp with an average G+C content of 66.5%, which is consistent with previously sequenced *P. aeruginosa* strains. It contains 6,052 putative genes (5,975 open reading frames [ORFs] and 77 RNA genes). Twelve genomic islands were predicted by IslandViewer analysis (13). Three of the genomic regions carrying putative islands distinguish RP73 from other *P. aeruginosa* reference genomes and correspond to regions of genome plasticity (14). Namely, the RP73 genome contains PAGI-9 (15) and plasmid pKLC102, which carries the *pil* gene cluster and *chvB* glucan synthetase (14). The RP73 genome also contains LESGI-4, which was identified in the Liverpool epidemic strain (16). More detailed analyses of the RP73 genome, including comparative analysis with other *P. aeruginosa* strains and identification of virulence factor-associated mutations, are in progress.

### Nucleotide sequence accession number. 

The complete RP73 genome sequence and annotation have been deposited at CCBJ/EMBL/GenBank under the accession number CP006245.1.
